# Extensive intragenomic variation in the internal transcribed spacer region of fungi

**DOI:** 10.1016/j.isci.2023.107317

**Published:** 2023-07-10

**Authors:** Michael J. Bradshaw, M. Catherine Aime, Antonis Rokas, Autumn Maust, Swarnalatha Moparthi, Keila Jellings, Alexander M. Pane, Dylan Hendricks, Binod Pandey, Yuanning Li, Donald H. Pfister

**Affiliations:** 1 Harvard University Herbaria and Department of Organismic and Evolutionary Biology, Harvard University, Cambridge, MA 02138, USA; 2 Department of Botany and Plant Pathology, Purdue University, West Lafayette, IN 47907, USA; 3 Department of Biological Sciences and Evolutionary Studies Initiative, Vanderbilt University, Nashville, TN 37235, USA; 4 School of Environmental and Forest Sciences, University of Washington, Seattle, WA 98195, USA; 5 Department of Entomology and Plant Pathology, North Carolina State University, Raleigh, NC 27695-7613, USA; 6 Department of Plant Pathology, North Dakota State University, Fargo, ND 58102, USA; 7 Institute of Marine Science and Technology, Shandong University, 72 Binhai Road, Qingdao 266237, China

**Keywords:** Molecular biology, Microbiology, Evolutionary biology

## Abstract

Fungi are among the most biodiverse organisms in the world. Accurate species identification is imperative for studies on fungal ecology and evolution. The internal transcribed spacer (ITS) rDNA region has been widely accepted as the universal barcode for fungi. However, several recent studies have uncovered intragenomic sequence variation within the ITS in multiple fungal species. Here, we mined the genome of 2414 fungal species to determine the prevalence of intragenomic variation and found that the genomes of 641 species, about one-quarter of the 2414 species examined, contained multiple ITS copies. Of those 641 species, 419 (∼65%) contained variation among copies revealing that intragenomic variation is common in fungi. We proceeded to show how these copies could result in the erroneous description of hundreds of fungal species and skew studies evaluating environmental DNA (eDNA) especially when making diversity estimates. Additionally, many genomes were found to be contaminated, especially those of unculturable fungi.

## Highlights

• The ITS region is the most prevalent barcode used in studies of kingdom Fungi
• There are multiple copies of the ITS region within the fungal genome
• ITS intragenomic variation and contaminated genomes are common in fungi
• Intragenomic variation can affect taxonomy, eDNA studies, and diversity estimates

## Graphical abstract

**Figure:**
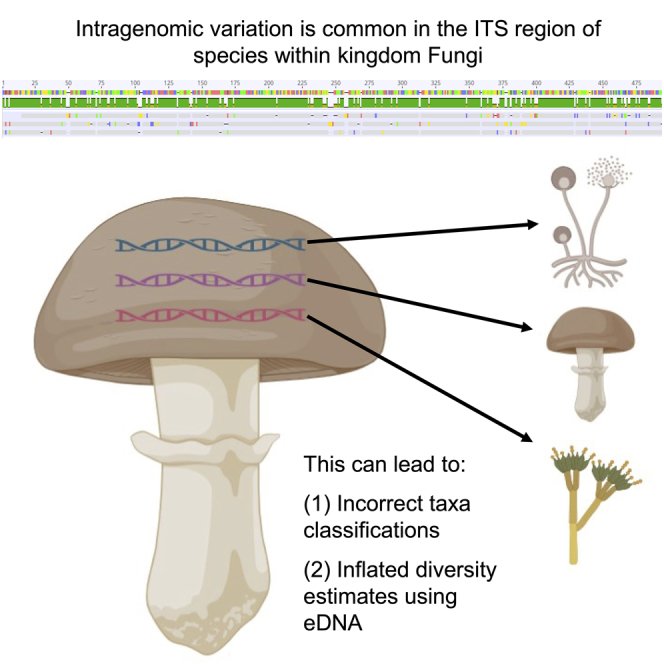

## Introduction

Fungi are one of the largest kingdoms among eukaryotes, containing an estimated 2.2–3.8 million species,^1^ but only ∼150,000 have been accepted so far. A key step in studying fungi, and for any downstream analyses, is species identification. Due to a dearth of diagnostic morphological characteristics and our inability to isolate or maintain many fungi in pure culture, accurate identification largely relies on the use of molecular markers. The nuclear ribosomal internal transcribed spacer (ITS) region of the rDNA array is the most prevalent marker used to identify fungi and is the primary fungal barcode to the Consortium for the Barcode of Life.^2^ The internal transcribed spacer region is also the most frequently applied marker in studies evaluating environmental DNA (eDNA).^3^ One of the main appeals of the ITS region is that each organism contains many paralogous copies^4^ allowing successful amplification of samples (such as eDNA and unculturable fungi) where quantities of DNA may be scant. Additionally, the unique combination of conserved areas of DNA encompassing highly variable DNA allows the ability to design robust and nearly kingdom-wide PCR primers.^5^ Considering that the ITS region was the first barcode commonly used for fungi, initial work using this region has led to many revelations regarding the generic and familiar classification of fungi. However, the multiple copies of ITS^4^^,^^6^^,^^7^^,^^8^ can sometimes vary, raising concerns on its broad usage throughout the kingdom.^9^^,^^10^

The multiple copies of the ITS region tend to be in clusters within the genome and are often homogenized by concerted evolution.^11^^,^^12^ Concerted evolution is not a definite phenomenon and mutations that are not homogenized can result in the formation of imperfect copies of functional genes and other intragenomic variation aberrations within the rDNA.^13^^,^^14^^,^^15^ Intraspecific variation in the ITS region is widespread within fungi^16^ and may cause ambiguous results when analyzing sequence data.^9^^,^^10^^,^^17^^,^^18^ In some cases, analyses of sequences from divergent ITS copies can result in the description of species, thus highlighting how use of rDNA alone can mislead taxonomic inference.^9^^,^^10^^,^^17^^,^^19^

Thanks to systematic sequencing efforts,^20^ fungi are one of the most densely genome-sequenced kingdoms of eukaryotes. Examination of fungal genomes offers new insight to study intragenomic variation.^10^^,^^21^ Here, we evaluated a genome assembly for each fungal taxon available on GenBank to determine the prevalence of intragenomic variation in kingdom Fungi, and the consequences of this variation on taxonomic conclusions, eDNA studies, and fungal diversity estimates.

## Results

### Data acquisition

In total, genome assemblies from 2414 taxa were mined and evaluated from GenBank (Data S1 and S2). The alignments generated are available at Dryad (https://doi.org/10.5061/dryad.g79cnp5t7). For approximately one-quarter of all the taxa evaluated (695/2414 assemblies), we were unable to locate an ITS sequence (ITS copies = 0). However, most of the taxa evaluated had one ITS copy (1080/2414). The remaining 641 taxa had two or more ITS copies.

### Intragenomic variation

**Figure 1:**
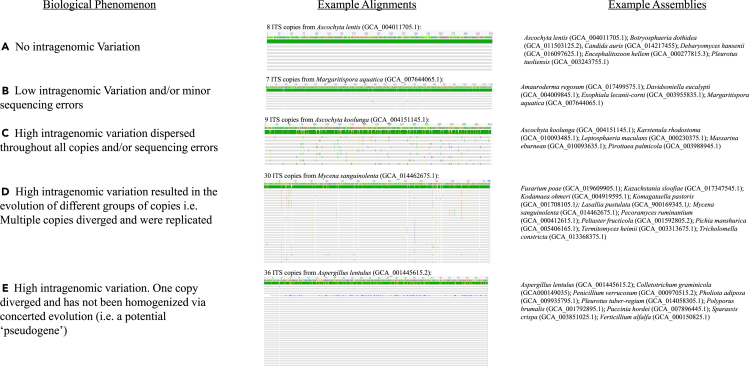
Common alignments observed when evaluating the ITS copies of fungi Example assemblies are provided for the different commonly observed alignments as well as hypotheses on their biological significance (A–E). Different colors in the alignment represent intragenomic variation.

641/2414 genome assemblies (∼27%) were found to have multiple ITS copies. The ITS copies were aligned, and five different phenomena were commonly observed (Figure 1). Of the 641 assemblies, 222 had 100% identical ITS copies (Figure 1A), 303 had low intragenomic variation (98–99.99% pairwise identity) (Figure 1B), and the remaining 116 assemblies had high variation (<98% pairwise identity) (Figures 1D and 1E). Highly divergent ITS copies, belonging potentially to “pseudogenes”, were found in 46 assemblies (Figures 1D and 1E); these copies have less than 93% sequence similarity with the other copies in the alignment. In total, ∼17% (419/2414) of the assemblies evaluated contained some level of intragenomic variation (pairwise identity <100%). The 17% intragenomic variation observed in the present study when analyzing all the assemblies is likely an underestimate as assemblies containing 0 or 1 ITS copy were coded as having no variation. In our analysis, when only multi-copy assemblies (ITS copies >1) were evaluated, ∼65% of the assemblies contained intragenomic variation. The variation observed can affect phylogenies differently. For example, random point mutations that may arise from sequencing errors or biological variation (Figures 1B and 1C) usually will have no effect in multiple alignment-based analyses. However, pseudogenes (Figure 1E) will usually form a unique clade.

Although we cannot exclude sequencing errors, the high coverage and low sequencing error rate for most of the sequencing technologies used for the assemblies evaluated^22^^,^^23^^,^^24^ suggest that the variation we observed among ITS copies was genuine. However, the sequencing technology, assembly methods, and technology read size can influence the data as discussed by Tedersoo et al.^25^ and Appendix D of Paoli et al.^10^ For example, assemblies constructed using long-read technology (PacBio, Oxford, etc.) tended to have largerer genomes (∼46 mbps [million base pairs]), higher GC percentages (48.11), more ITS copies (14.52) and less intragenomic variation (pairwise identity = 99.11) compared to short-read technology (Illumina, sanger, etc.) (genome size = ∼37 mbps; GC percentage = 47.3; ITS copies = 1.49; pairwise identity = 96.61) (Data S2). The most accurate data undoubtedly occurs when both long and short-read technologies are used in tandem (genome = ∼44 mbps; GC percentage = 46.01; ITS copies = 9.43; pairwise identity = 98.99) (Data S2).

### Contamination

Multiple contaminated assemblies from the 2414 genomes were identified (Data S3). The contaminants were mostly identified from fragmented ITS copies that were GenBank nblasted and found to align with accessions of a different taxon than the assembly was designated. After noting that many of the contaminated genomes were from obligate parasitic fungi that cannot be cultured, we proceeded to evaluate intensely the assemblies of two commonly studied unculturable pathogens (Erysiphaceae, powdery mildews, and Pucciniales, rust fungi) to determine how common contamination is in unculturable full genome assemblies. In total, 12/35 of the taxa evaluated from Erysiphaceae and Pucciniales were contaminated. Another observation is that assemblies may be derived from multiple strains of the same species. For example, *Nosema bombycis* (GCA 000383075.1) has 9 ITS copies with a 95% pairwise identity among the copies. However, when GenBank blasted, the different copies aligned with different strains. A similar situation was found in *Suillus spraguei* (GCA016800925.1) where 4 copies aligned 100% with the ITS of *Suillus spraguei* voucher ACAD21063F (OL741513), whereas two other copies aligned 100% with *Suillus spraguei* strain EM44 (OL685247). Another possibility is that the authors of the accessions on GenBank sequenced the different ITS copies in the genome and reported these different “copies” as “strains.” This phenomenon was also observed and discussed by Stadler et al.^15^ who discovered 5 deviating ITS copies (one of which was a pseudogene) of *Hypomontagnella monticulosa*.

## Discussion

Our study reveals that fungi exhibit intragenomic variation in the form of nucleotide substitutions, deletions/insertions, and likely “pseudogenes” which we show can be impacting taxonomic, eDNA, and diversity estimate studies. The high intragenomic variation in the ITS rDNA observed in the present study can be attributed to the divergence of multiple copies that have not been homogenized through concerted evolution or similar forces. Evaluating full genome assemblies can give an accurate representation of intragenomic variation^10^^,^^15^; as such the ITS intragenomic variation reported here is likely indictive of the true intragenomic variation in kingdom Fungi. In our analysis, when only assemblies with ITS copies >1 were evaluated, ∼65% of the assemblies contained intragenomic variation. However, when only high-quality assemblies using both long- and short-read technology are considered, variation is still observed in a high percentage of the taxa evaluated (49.7% [90/181]), and in a similar proportion to that reported for all multi-copy assemblies (∼65% variation in assemblies containing >1 copy). The 49.7% is likely more indictive of the true proportion of ITS intragenomic variation of taxa in kingdom Fungi. Lindner et al.^16^ estimated that rDNA intragenomic variation was widespread, yet rare in fungi, with polymorphisms to exist in ∼3–5% of taxa. This is a considerable underestimate from the data shown in the current study. The intragenomic variation data presented are consistent with the data from Paloi et al.^10^ and Stadler et al.^15^ It should be noted that there were differences in intragenomic variation between all the taxonomic groupings evaluated (Data S2). Organisms within the earlier diverging taxa (Chytridiomycota, Mucoromycota, and Zoopagomycota) tended to have the most intragenomic variation.

Fungi were estimated to have between 14 and 1442 ITS copies in their genomes based on an in silico read depth approach.^4^ Although mining assemblies can give an accurate representation of intragenomic variation, it does not give reliable data of ITS copy numbers.^10^ The effect of the different sequencing technologies on ITS copy number is discussed thoroughly by Tedersoo et al.^25^ and in Appendix D of Paoli et al.^10^ Briefly, the impact of assembly programs on copy number can, in part, be due to the “stacking” function of the assembly pipelines. For example, similar data can be stacked on top of each other (under certain identity thresholds), essentially masking copy numbers. We hypothesize that this “stacking function” is likely the cause for the massive amount of single-copy ITS regions observed throughout the dataset, which is why they were not included in our main intragenomic variation analyses. When only assemblies with ITS copies >1 were evaluated, the number of copies ranged from 2 to 528 with an average of 10.9. However, when only long-read technology was considered, the average ITS copy number was 14.52, which is likely the most accurate calculation of copy number. Even so, this is a vast underestimate of the average of 133 copies presented by Lofgren et al.^4^

The evolution of extremely divergent ITS copies (i.e., likely “pseudogenes”) is common throughout fungi (Figure 1). In our evaluation of the current data, we found instances in which it is possible that species were described based on a divergent ITS copy. For example, *Candida viswanathii* (GCA003327735) has 5 ITS copies that align 100% with *Candida viswanathii* accessions from GenBank and 4 copies that align 99.7% with the type material of *Candida pseudoviswanathii*. Unsurprisingly, Ren et al.^26^ described *C. pseudoviswanathii* based primarily on ITS sequences. Taxonomic conclusions regarding these species should not be made until the type material of *C. pseudoviswanthii* and *C. viswanathii* can be further evaluated with additional genetic markers. Similar examples in yeasts with divergent ITS copies are discussed in Sipiczki^27^ and Sipiczki.^28^ Other examples of accessions/species that need to be evaluated further include *Mucor circinatus* (GCA_016758965.1), the ITS copies of which align with different *Mucor* spp. i.e., *Mucor plumbeus* and *M. mucedo*, and *Taphrina wiesneri* (GCA_005281515.1), the copies of which align with type material of *T. wisneri* and type material of *Taphrina confusa*. Similar cases likely exist in the dataset and we encourage researchers to further mine the data to locate doubtful species that were potentially described based on divergent ITS copies. Further critical analyses should also be conducted to see if any of these circumstances are examples of hybridization between different fungi. Only 2414 of the ∼150,000 taxa accepted from kingdom fungi were analyzed here and as such intragenomic variation could have led to the description of hundreds of erroneous species. Interestingly, the “pseudogenes” analyzed from the present study often contained multiple mismatches with the common fungal primers (ITS1, ITS4, and/or ITS5) from White et al.,^5^ and are often in lower proportion to the other ITS types (Figure 1E).^16^ This likely explains why they are not commonly amplified in PCR. Additionally, when other primers are used, or non-specific binding occurs, it could lead to new species being described based on a divergent ITS copy as was reported by Harrington et al.^17^ A similar phenomenon in *Fusarium* was noted where two highly divergent ITS 2 “types” were observed, of which, only the “major ITS2 type” was able to be sequenced with conserved primers. When the authors developed specific primers, they were able to anneal and amplify the other ITS type.^29^ Different primers can anneal to different ITS copies and, as such, the primers used could be artificially skewing the phylogenetic relationships among certain fungal lineages.

Caution should be taken when describing species based predominantly on differences in the ITS region without corresponding secondary barcodes and/or morphological, ecological, and chemical data.^30^ The effect of ITS copies likely has a large impact on eDNA studies that rely on ITS data. Kõljalg et al.^3^ proposed the term “species hypothesis,” for taxa discovered through ITS analyses that grouped together in different similarity thresholds ranging from 97 to 99%. The UNITE platform^31^ variously delimited species hypothesis at 97–100% similarity based on intraspecific ITS variability. Additionally, the GlobalFungi database^32^ classified ITS sequences according to the closest UNITE species hypothesis and a 98.5% similarity threshold. Evaluating our 641 assemblies that contained >1 ITS copy, at a 97% “species hypothesis” threshold, we could describe an additional 15% (93/641) species, at a 98% threshold, an additional 18% (116/641) species, and at a 99% threshold, an additional 27% (171/641) species. Similarly, Lindner and Banik^19^ showed how the use of a 95% threshold for *Laetiporus* species descriptions based on the ITS region could artificially result in over twice the number of described species due to intragenomic variation of ITS copies. It is likely that the use of amplicon sequence variants evaluating the ITS region in eDNA studies is also being impacted by intragenomic variation. Lücking et al.^33^ found that sequencing errors in some full genome technology can contribute to increased biological diversity estimates. As such, using eDNA data from the ITS region to establish diversity estimates^34^ could be vastly overestimating the number of fungi.

During this study, a high number of assemblies were determined to be contaminated with non-target fungi (Data S3). This was especially the case with unculturable fungi (at least 82% of the taxa in the Erysiphaceae were found to contain contaminants and at least 12.5% of the taxa in the Pucciniales were contaminated). Similar to the current study, Vaghefi et al.^35^ noticed the high contamination among Erysiphaceae genomes and recommended that assemblies of taxa within the order be treated as “eDNA,” recognizing that the sequences from leaf tissue and surfaces were likely to include other organisms in the environment. Future research evaluating full genome phylogenies should consider removing unculturable fungi from their analyses or methodically checking them for contamination by blasting the assemblies with common contaminate DNA from multiple regions (blasting solely the ITS region is not sufficient as many assemblies do not contain an ITS region). Removing contaminated assemblies from datasets will undoubtedly improve phylogenetic inference.

The issue of misidentifications, contaminations, and assemblies that contain multiple strains cannot be discounted and is likely a common occurrence within fungi (even in pure cultures).^10^^,^^35^^,^^36^ We hypothesize that the high amount of DNA required for full genome sequences may lead to these intra/inter species contamination issues through the accumulation of multiple individuals for processing. It is possible that sequencing multiple strains/taxa could be impacting other molecular statistics such as genome size. For example, some obligate, unculturable fungi have been reported to represent the largest, repeat rich, genomes.^37^ In our dataset, a potential example of this phenomenon can be observed with the assembly from *Austropuccinia psidii* (GCA 902702905.1). This is the third-largest genome in our dataset (Data S1). It contains 10 ITS copies; one copy is likely a pseudogene and the remaining 9 fall into two genotypes. One genotype aligns 100% with *Puccinia psidii* isolate UY217 (EU348742) and the other aligns 100% with *Puccinia psidii* isolate SZ2 (EU071045). In this scenario, it is possible that the genome contains multiple strains that artificially increase the genome size and repetitive regions. Alternatively, in the case of this rust, we could be observing two parental genotypes.

The ITS region is widely accepted as the universal barcode for fungi.^2^ Our analyses show that ITS intragenomic variation is common throughout kingdom Fungi (Data S1), a finding with wide implications for taxonomic assignments, eDNA analyses, and fungal diversity estimates. Future research evaluating the ITS region should consider the data generated (available at Dryad: https://doi.org/10.5061/dryad.g79cnp5t7) to ascertain whether intragenomic variation could be skewing research results. Internal transcribed spacer region data have been analyzed for fungi for over 30 years and these data should not be set aside, however, taxonomic conclusions using ITS data should be accompanied by secondary barcodes and/or morphological and ecological data.^38^^,^^39^ Additionally, DNA-based typifications^30^^,^^40^ should not be done solely with ITS. Future research should further deduce the role that intragenomic variation can play on eDNA studies, especially regarding diversity estimates. Other single-copy markers should be evaluated and compared to ITS data in eDNA studies to ascertain the effect of intragenomic variation. Additionally, the data presented could be mined further to answer a range of molecular biology questions including the substitution rate and most common intragenomic mutations occurring in the ITS rDNA region. Sequencing genomes is becoming easier and more affordable. We recommend future taxonomic research to consider taking a taxogenomic approach and eDNA studies to use other single-copy markers to circumvent the issues presented here.

### Limitations of the study

A major limitation of the study is the effect of sequencing technology on the results, especially in regard to sequencing errors. Additionally mining genomes does not give reliable data of ITS copy numbers. As such, we were unable to determine the proportion of each of the different variant copies within a genome. Having said that, we believe the intragenomic variation data are genuine and a detailed discussion of the potential impact of the sequencing technology can be found in the results and discussion sections. Additionally, considering that the present study was accomplished bioinformatically, none of the results were verified in the lab.

## STAR★Methods

### Key resources table

**Table:** 

REAGENT or RESOURCE	SOURCE	IDENTIFIER
**Deposited data**		

ITS genome alignments	Dryad	https://doi.org/10.5061/dryad.g79cnp5t7
Raw Data	This paper	Data S1, S2, and S3

**Software and algorithms**		

GenBank	Sayers et al.^41^	GenBank Overview (nih.gov)
Geneious version 2021.2.2	Geneious	https://www.geneious.com
R (v. 3.31)	R Foundation for Statistical Computing	

### Resource availability

#### Lead contact

Further questions should be directed to Dr. Michael Bradshaw (mbradshaw@fas.harvard.edu).

#### Materials availability

This study did not generate new unique reagents.

### Experimental model and subject details

This work has not involved the use of human subjects or samples, nor has it used experimental models that require reporting of experimental model and subject details.

### Method details

Data were mined from at least one genome assembly of every fungal species from September-December of 2021 on GenBank.^41^ Data mining was accomplished by extracting the multiple ITS copies from a given assembly and then aligning and analyzing the extracted copies for variation. Detailed methods are as follows.

(1) A list of all taxa with publicly available assemblies was compiled.
(2) For each taxon, GenBank’s nucleotide database was searched for a fully annotated ITS region.
(3) The GenBank accession number determined from (2) was GenBank blasted (blastn) to ensure the taxon was identified correctly.
(4) The ITS region from (2) was trimmed to include only nucleotides present in the ITS1+5.8S+ITS2 region.
(5) A genome assembly was chosen for each fungal species on GenBank. If multiple assemblies for a given taxon were available, the assembly with the smallest number of scaffolds/contigs was evaluated first.
(6) A genome assembly was GenBank blasted (blastn) with the trimmed ITS region. For example, in Data S1, column A (‘Assembly Reference’) was GenBank blasted with column E (‘GenBank Accession Number of ITS Region used to blast assembly’); if no ITS region was located or if it was very fragmented other assemblies were checked.
(7) The results of the assembly blast were downloaded into Geneious version 2021.2.2 and aligned.
(8) ITS copies from the genome assembly that were ∼ >50 bases shorter than the length of the ITS region determined in step (4) were discarded to eliminate short contigs and to keep the data consistent.
(9) Alignments for these taxa are available on Dryad (https://doi.org/10.5061/dryad.g79cnp5t7) in both a .geneious and .fasta file format.
(10) The number of ITS copies in the assembly, identical site % and pairwise identity % among the different copies were calculated in Geneious and recorded.
(11) The ITS accessions used to blast the assemblies were downloaded into Geneious and their GC content was recorded.
(12) The remaining data from the assemblies were recorded from GenBank (Taxa ID, assembly method, sequencing technology used, genome coverage, contigs, scaffolds, assembly GC content (%), assembly release date, and genome size).

### Quantification and statistical analysis

All data analyses were conducted in the software R v. 3.31.

## Data Availability

• The data is available in Data S1, S2, and S3 as well as through Dryad. DOIs are listed in the key resources table. • This paper does not report original code. • Any additional information required to reanalyze the data reported in this paper is available from the lead contact upon request.

## Supplemental information

Data S1. Raw data compiled in the current study from the ITS genome alignments and GenBank, related to STAR Methods

Data S2. Averages of the different variables evaluated from Data S1, related to STAR Methods

Data S3. Genome assemblies found to be contaminated in the current study In each of these assemblies, at least one of their ITS copies, aligned with accessions of a different taxon than the assembly was designated. Contaminations were determined from their ITS genome alignments and GenBank, related to STAR Methods.
